# PEG-mediated transformation: a tool for random integration or targeted gene replacement in *Colletotrichum camelliae*

**DOI:** 10.3389/fmicb.2026.1814272

**Published:** 2026-04-24

**Authors:** Fang-Fang Huang, Yu-Huan Xie, Qun Hu, Mei-Xi Huang, Ling-Zhi Zhang, Zhong-Hua Liu, Juan Li, Jian-An Huang, Li-Gui Xiong

**Affiliations:** 1 Key Laboratory of Tea Science of Ministry of Education, Hunan Agricultural University, Changsha, Hunan, China; 2 National Research Center of Engineering Technology for Utilization of Functional Ingredients from Botanicals, Hunan Agricultural University, Changsha, Hunan, China; 3 Yuelushan Laboratory, Changsha, Hunan, China; 4 National Key Laboratory for Tea Plant Germplasm Innovation and Resource Utilization, Changsha, Hunan, China

**Keywords:** *C. camelliae*, PEG-mediated transformation, random insertional mutagenesis, targeted gene deletion, cutinase gene

## Abstract

**Introduction:**

*Colletotrichum camelliae* is a highly virulent fungal pathogen responsible for anthracnose in tea plants, which poses a major threat to tea production. The development of a reliable genetic transformation system for *C. camelliae* is a fundamental prerequisite for elucidating its molecular pathogenic mechanisms and advancing functional genomic research in this pathogen.

**Methods:**

In this study, polyethylene glycol (PEG)-mediated protoplast transformation was optimized for genome modification in *C. camelliae*. Protoplasts were efficiently generated through enzymatic digestion of young mycelia using a mixture containing 20 mg/mL driselase, 15 mg/mL lyticase, and 15 mg/mL snailase at 28 °C for 3 h. The optimized protocol was validated by introducing the plasmid pKD7-RED, carrying a red fluorescent protein (RFP) coding sequence, into the fungal genome, and further applied for targeted disruption of the cutinase gene via PEG-mediated transformation coupled with homologous recombination.

**Results:**

The optimized enzymatic digestion protocol yielded a high quantity of viable protoplasts, which was critical for successful transformation. Fluorescence microscopy confirmed stable RFP expression across five consecutive generations, during conidial germination on hydrophobic surfaces, and throughout the infection process on tea leaves, demonstrating the heritability and stability of transgene expression. Furthermore, targeted disruption of the cutinase gene showed that it had no effect on the pathogenicity of *C. camelliae* under wounded inoculation conditions.

**Discussion:**

These findings demonstrate that PEG-mediated transformation is an efficient and versatile approach for both random insertional mutagenesis and targeted gene deletion in *C. camelliae*. This established genetic manipulation system lays a solid technical foundation for future functional genomic studies of *C. camelliae* pathogenicity and the development of novel strategies for tea anthracnose control.

## 1 Introduction

*Colletotrichum* species are widespread phytopathogenic fungi known to cause anthracnose in numerous economically important crops, including cucurbits, apples, grapes, and soybeans, drawing extensive research attention (da Silva et al., 2020; Liu et al., 2024b; Yan et al., 2015; Boufleur et al., 2021). *C. sinensis*, as a major economic crop cultivated for tea production, is susceptible to anthracnose caused by *Colletotrichum* spp., under warm and humid environmental conditions. This disease significantly compromises plant health, is prevalent in Bangladesh, China, Hawaii, Japan, India, Indonesia and Sri Lanka, and poses a serious threat to the sustainable development of the global tea industry (Pandey et al., 2021). Current management strategies for preventing and controlling anthracnose include agricultural practices, chemical control, biological agents, and integrated approaches (Liu et al., 2022; Rao, 2021). Different cultivars of tea plants exhibit varying levels of susceptibility to anthracnose, making the breeding of resistant varieties an effective preventive strategy. *C. camelliae* overwinter in tea plant tissues and soil; under favorable temperature and humidity conditions in spring, the pathogen reproduces and infects the leaves. The conidia produced at lesion sites facilitate secondary spread of the disease, highlighting the importance of thorough garden sanitation during winter as a crucial control measure (Liu et al., 2022). Supplementary management of anthracnose can also be effectively accomplished through agronomic practices such as weeding, pruning, and rational fertilization (Rao, 2021). Chemical control methods involve the application of fungicides, such as carbendazim and chlorothalonil. It is essential to adjust fungicide concentrations based on the disease stage and to avoid the prolonged use of a single fungicide to prevent the development of resistance. In contrast, biological control offers a more environmentally friendly alternative that utilizes biocontrol microorganisms for effective disease management (Liu et al., 2022).

Investigating pathogen-plant interactions to identify previously unknown mechanisms of interaction or potential antipathogen targets may offer an alternative strategy for designing novel antifungal compounds. Conserved effector proteins serve as potential fungicidal targets and can be leveraged to achieve antifungal effects (Liu et al., 2024a). While the genome of *C. camelliae* has been sequenced (Kong et al., 2023), most functional genes, including its effector proteins, have not been thoroughly analyzed and require further investigation. Consequently, the development of molecular genetic tools to explore the biological functions of these target genes is essential. A robust genetic transformation system serves as a fundamental tool for the functional characterization of virulence-related genes. Commonly employed methods for genetic transformation in filamentous fungi include polyethylene glycol (PEG)-mediated protoplast transformation, *Agrobacterium tumefaciens*-mediated transformation (ATMT), electroporation, particle bombardment, restriction enzyme-mediated integration (REMI), and shock wave-mediated transformation, etc (Bai and Lu, 2023). Among these, PEG-mediated transformation is widely favored due to its simplicity, high efficiency, low equipment requirements, and capacity for co-transformation of multiple DNA fragments. It remains the most commonly used technique for fungal genetic engineering (Bai and Lu, 2023; Liu and Friesen, 2011). This method has been successfully applied in diverse fungal systems, including the plant endophytic fungus *Alternaria oxytropis* (Lu P. et al., 2021; Lu W.-Y. et al., 2021), the biocontrol fungus *Clonostachys rosea* (Chen et al., 2021), the human opportunistic pathogen *Aspergillus flavus* (Jiang et al., 2019), as well as phytopathogens such as *C. falcatum, Fusarium graminearum, Magnaporthe grisea, F. oxysporum* f. sp. *vasinfectum, Sporisorium reilianum*, and *C. fructicola* (Amalamol et al., 2022; Ding et al., 2009; Li et al., 2004; Xu et al., 2012; Ning, 2009; Han et al., 2016).

In our research, we systematically optimized the key parameters influencing transformation efficiency and subsequently explored the application of PEG-mediated transformation as a versatile tool for both random insertional mutagenesis and targeted gene disruption in *C. camelliae*. In addition, building upon the PEG-mediated transformation system, we successfully developed a gene knockout system by utilizing the principle of homologous recombination and employing split-marker technology. This approach enabled us to generate cutinase gene deletion mutants, thereby demonstrating its utility for functional genetic studies.

## 2 Materials and methods

### 2.1 Strain and plasmid information

The *C. camelliae* strain F5 was isolated from tea leaves and grown at 28 °C on potato dextrose agar (PDA). The plasmid pKD7-RED was generously providedby Professor Lu. This vector is designed for fluorescent fusion protein expression in filamentous fungi, harboring the *histone H3* promoter and the *DsRED2* reporter gene (Li et al., 2012).

### 2.2 Geneticin (G418) sensitivity assay in *C. camelliae* F5

Sensitivity to G418 was evaluated using a gradient plate assay. Briefly, activated *C. camelliae* F5 cultures were inoculated onto PDA plates supplemented with 0, 40, or 80 μg/mL G418 and incubated at 28 °C for 7 days, with three biological replicates per condition. Colony growth was recorded, and colony areas were measured using ImageJ.

### 2.3 Protoplast preparation

Protoplast isolation was performed using young mycelia derived from conidial germination as the starting material. The cell wall was enzymatically digested with a mixed hydrolytic enzyme solution containing driselase, lyticase, and snailase, prepared in 0.7 M NaCl. To obtain young mycelia of *C. camelliae* F5, the strain was initially cultured in potato dextrose broth (PDB) at 28 °C for 5 days to harvest conidial pellets. The pellets were resuspended and washed in sterile distilled water to yield a conidial suspension at a final concentration of 1 × 10^7^ /mL. This suspension was inoculated into fresh PDB medium at a ratio of 1:100 (v/v) and incubated overnight to generate young, actively growing mycelia. The enzymatic hydrolysis of the cell wall was carried out at 28 °C and 80 rpm. After enzymatic hydrolysis, protoplast was collected by double-layer sterile lens paper filtration. After centrifugation at 4,000 rpm and 4 °C for 15 min, half of the supernatant was discarded, and an appropriate volume of STC buffer (200 g/L sucrose, 10 mM Tris-HCl pH 8.0, 5.540 g/L CaCl_2_) was added to adjust the NaCl-STC volume ratio to 1:1 for gradual osmotic adaptation, which effectively minimizes protoplast loss and lysis risk. After a brief equilibration period, the mixture was centrifuged again at 4,000 rpm and 4 °C for 15 min to remove residual impurities. The supernatant was then fully discarded, and the pellet was gently resuspended in pure STC buffer, followed by adjusting the protoplast concentration to 10^8^ /mL.

### 2.4 PEG-mediated transformation

A volume of 100–200 μL of protoplast suspension was mixed with the target DNA fragment or plasmid (2 μg), gently vortexed, and incubated at room temperature for 25 min to allow DNA uptake. Subsequently, 0.5 mL of PTC solution (STC supplemented with 40% w/v PEG 4000) was added, followed by an additional 15 min incubation at room temperature. Another 0.5 mL of PTC solution was then added, and the mixture was incubated for a further 10 min to complete the transformation process. The transformed protoplasts were thoroughly mixed with pre-cooled TB_3_ regeneration medium (200 g/L sucrose, 6 g/L casein acid hydrolysate, 6 g/L yeast extract, cooled to a touchable temperature) and poured into Petri dishes. After overnight incubation at 28 °C, the regenerated colonies were overlaid with PDA medium containing 80 μg/mL G418 for selection of transformants. Resistant colonies were subsequently transferred to fresh PDA plates supplemented with 80 μg/mL G418 for further characterization.

### 2.5 Pathogenicity assay

Tea leaves were surface-sterilized with 10% (v/v) sodium hypochlorite for 3 min, rinsed extensively with sterile distilled water, and wounded manually at symmetrical positions relative to the leaf veins on the abaxial side. Activated *C. camelliae* strains were inoculated onto the wound sites. Following inoculation, the leaves were placed in a humidified chamber and incubated at 28 °C. Disease development was monitored over time to assess pathogenicity.

### 2.6 Phenotypic characterization of fluorescent strains

Mycelial growth rate: *C. camelliae* strains were activated on PDA plates, and mycelial plugs were transferred to the center of fresh PDA plates in triplicate. Plates were incubated at 28 °C for 7 days, after which colony areas were calculated.

Conidiation assay: Activated wild-type (WT) strain *C. camelliae* F5 and positive transformants were individually inoculated into PDB medium and cultured at 28 °C with shaking at 200 rpm for 5 days. Mycelia were removed by filtration through sterile lens cleaning paper, and the filtrate was collected in 50 mL centrifuge tubes. Conidia were pelleted by centrifugation at 8,000 rpm for 10 min, resuspended in sterile distilled water, and counted using a hemocytometer to determine conidial yield. All experiments were conducted in triplicate.

### 2.7 Observation of conidial germination and infection process

Conidial germination: Conidial suspensions were adjusted to a concentration of 1 × 10^6^ /mL. A 10 μL aliquot was placed on hydrophobic glass slides and incubated under humid conditions at 28 °C for 24 h. Germination and appressorium formation were observed at 0, 2, 4, 6, 8, 10, 12, and 24 h using a Zeiss fluorescence microscope.

Infection process: Inoculation procedures followed those described in Section 2.5. Leaf samples were collected at 12, 24, 48, and 56 h post-inoculation and examined microscopically using a Zeiss fluorescence microscope to monitor fungal infection dynamics.

### 2.8 Gene knockout

**Figure 1:**
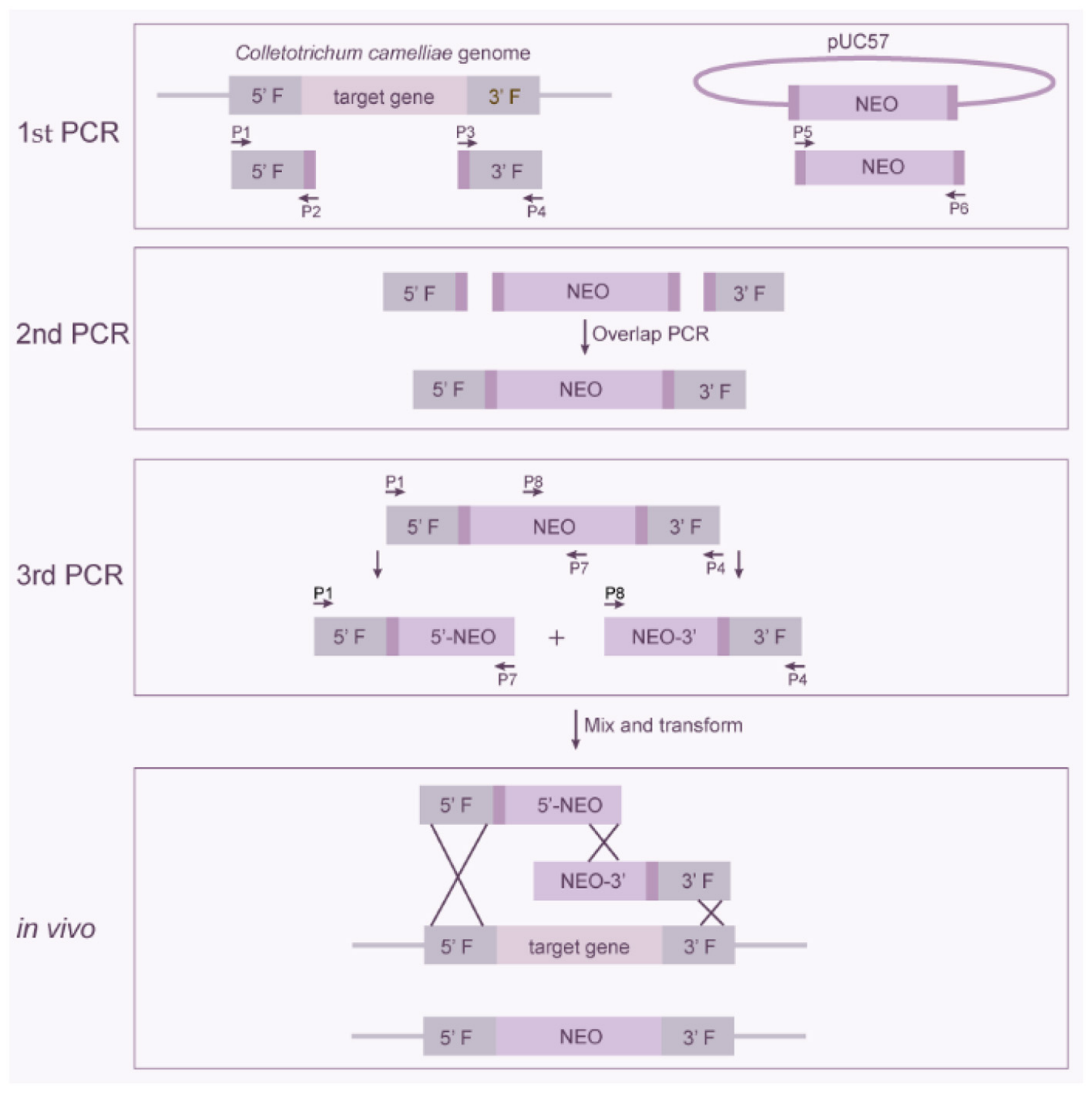
The workflow of gene knockout.

Gene knockout was achieved through homologous recombination, employing a split-marker PCR-based strategy in conjunction with PEG-mediated protoplast transformation. The knockout construct was produced through three consecutive rounds of PCR and was applied in genetic transformation to create gene disruption mutants (Figure 1). The detailed steps for fragment construction are outlined below:

(1) First-round PCR: Amplification of the left (upstream) and right (downstream) homologous flanking regions of the target gene, along with the selectable marker cassette (neomycin phosphotransferase gene, *neo*, under control of the *gpda* promoter and *trpC* terminator). Primers were designed to amplify 1.5–2.0 kb sequences upstream and downstream of the target gene. The primers were designed to include a 20 bp homologous sequence corresponding to the resistance gene cassette. Using genomic DNA isolated from *C. camellia* F5 as the template, the homologous arm sequences flanking the target gene were amplified and cloned. The resistance gene cassette was synthesized by Genscript Biotech Co., Ltd. Subsequently, primers were designed based on the pUC-57 plasmid—provided by the company and containing the resistance gene cassette sequence—to amplify the resistance gene cassette.
(2) Second round of PCR: Fusion of the homologous arms with the resistance gene cassette. The PCR products generated in step (1) served as templates for fusion PCR, enabling the ligation of the target gene's homologous arms to the resistance gene cassette.
(3) Third round of PCR: Amplification of knockout fragments. Using the fused product from step (2) as the template, specific primers were designed to amplify two distinct knockout fragments. Each fragment consisted of approximately two-thirds of the selectable marker gene and one homologous arm, intended for targeted gene replacement.

### 2.9 Identification of positive transformants

Identification of fluorescently labeled strains: Individual colonies capable of growth on G418-containing selective medium were examined for fluorescence emission using a Zeiss fluorescence microscope. Strains exhibiting red fluorescence were subcultured up to the fifth generation to assess fluorescence stability. Additionally, the presence of the *neo* gene was confirmed via PCR amplification using the following primer pair: *neo*_F: ACCACCAAGCGAAACATC; *neo*_R: TATCACGGGTAGCCAACG.

Identification of gene knockout mutants: Positive transformants were identified through combined antibiotic resistance screening and PCR validation. Genomic DNA was extracted from colonies growing on G418-supplemented medium and subjected to PCR analysis to confirm the integration of the resistance gene and knockout fragments, as well as the absence of the target gene fragment. Transformants that tested positive for both the resistance and knockout fragments but negative for the target gene fragment were classified as successful gene knockout candidates.

## 3 Results

### 3.1 Sensitivity of *C. camelliae* F5 to G418

The plasmid pKD7-RED used for genetic transformation carries the eukaryotic resistance selection marker gene *neo*, which encodes aminoglycoside -phosphotransferase and confers resistance to G418 in transformants. To establish an effective and specific screening protocol for positive transformants, it was essential to determine the sensitivity of the wild-type (WT) strain to G418 and identify the minimum inhibitory concentration (MIC) that completely suppresses WT growth. This step was crucial to prevent false-positive selections while ensuring the survival of true transformants.

**Figure 2:**
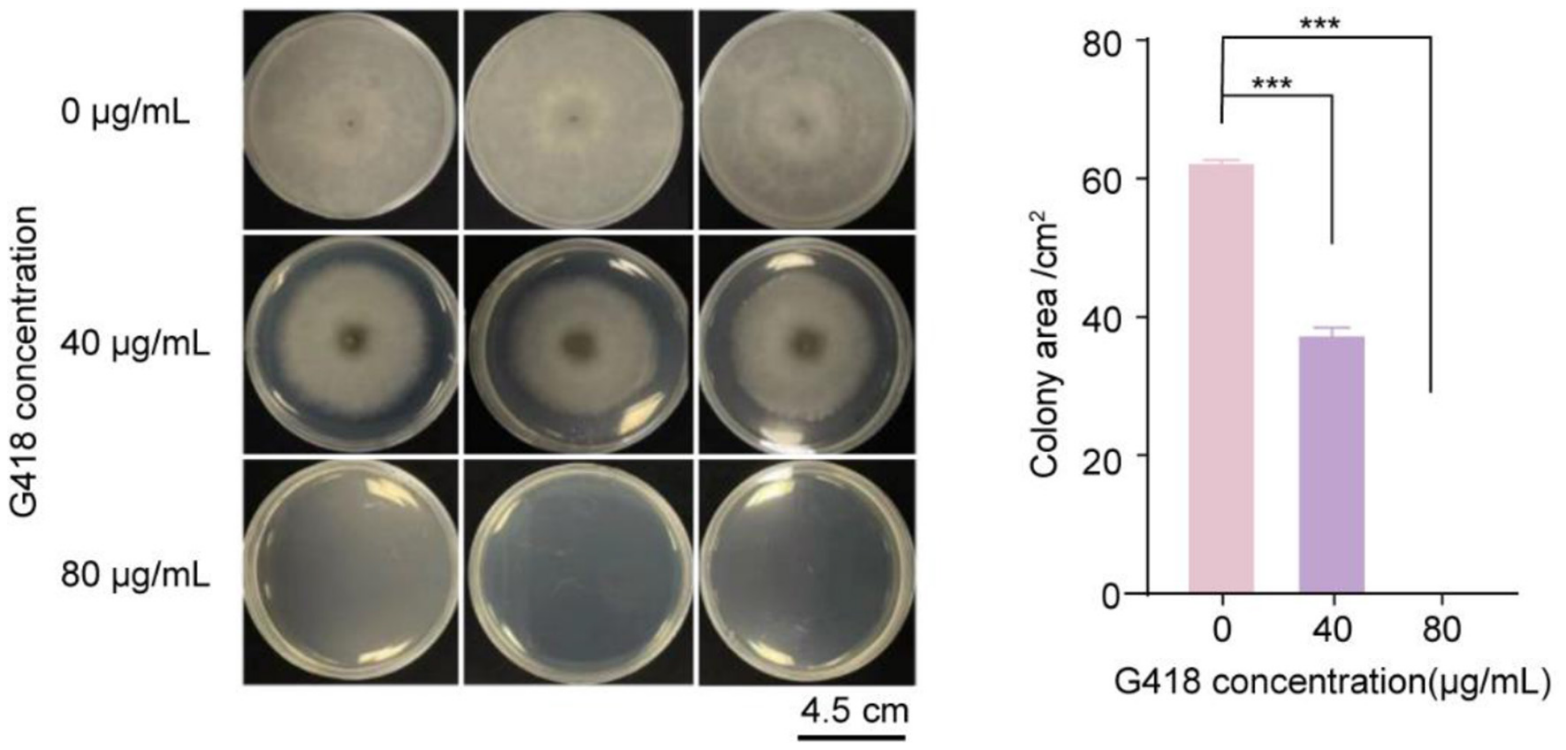
Sensitivity of *C. camelliae* F5 to G418. (*** means *p* < 0.001, two-tailed *t-*test).

The results of the sensitivity assay were shown in Figure 2. G418 at 40 μg/mL significantly inhibited the growth of *C. camellie* F5 compared to the control group (*p* < 0.001). In contrast, no visible colonies were formed by *C. camellie* F5 on PDA medium containing 80 μg/mL G418, and mycelial growth was entirely suppressed. Therefore, 80 μg/mL G418 was determined as the optimal concentration for selection of positive transformants following genetic transformation of *C. camelliae* F5. This concentration effectively eliminates residual wild-type background while applying sufficient selective pressure for transformants expressing the *neo* resistance marker, thus ensuring the accuracy and reliability of screening outcomes.

### 3.2 Protoplast preparation

Protoplast yield and viability—key quality indicators—directly affect the efficiency of downstream genetic transformation. Two major factors influence protoplast preparation efficacy: the composition of the enzymatic hydrolysis system and the duration of hydrolysis. Accordingly, this study first optimized the enzyme combination and subsequently refined the hydrolysis time to obtain protoplasts with high yield and viability, thereby supporting the development of a robust genetic transformation system for *C. camelliae* F5.

**Figure 3:**
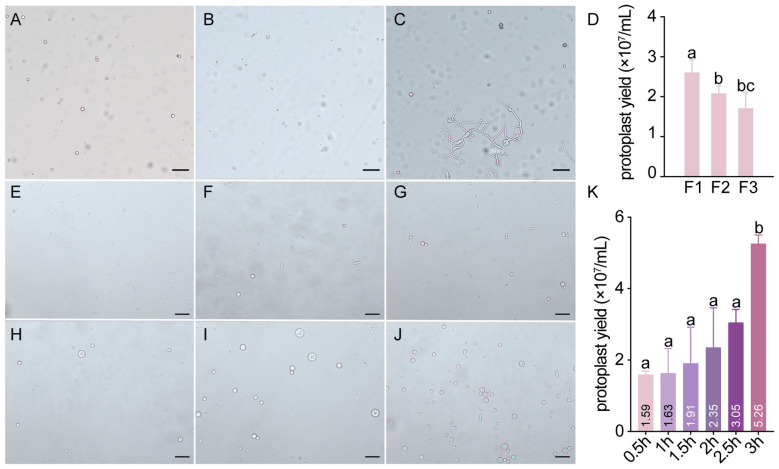
Optimization of protoplast preparation. **(A–C)** Protoplast prepared using different enzymatic formulas, **(A)** Formulation 1 (20 mg/mL driselase, 15 mg/mL lyticase, 15 mg/mL snailase), **(B)** Formulation 2 (30 mg/mL driselase, 10 mg/mL lyticase, 10 mg/mL snailase), **(C)** Formulation 3 (40 mg/mL driselase, 5 mg/mL lyticase, 5 mg/mL snailase); **(D)** Protoplast yield of different formulations (F1: Formulation 1, F2: Formulation 2, F3: Formulation 3, different letters indicate significant differences, *p* < 0.05, one-way ANOVA + LSD test) **(E–K)** Protoplast prepared in formula 1 at different times, **E:** 0.5 h, **F:** 1 h, **G:** 1.5 h, **H:** 2 h, **I:** 2.5 h, **J:** 3 h; **(K)** Protoplast yield in formula 1 at different times (different letters indicate significant differences, *p* < 0.05, one-way ANOVA + LSD test); Scale bars: **(A–C, E–J)** = 50 μm.

To identify the optimal enzyme formulation, preliminary experiments were conducted to evaluate the hydrolysis efficiency of ternary enzyme mixtures composed of driselase, lyticase, and snailase. Three formulations with varying concentration ratios were tested: formulation 1 (20 mg/mL driselase, 15 mg/mL lyticase, 15 mg/mL snailase); formulation 2 (30 mg/mL driselase, 10 mg/mL lyticase, 10 mg/mL snailase); and formulation 3 (40 mg/mL driselase, 5 mg/mL lyticase, 5 mg/mL snailase). Young mycelia were subjected to enzymatic digestion at 28 °C and 80 rpm for 2 h, and hydrolysis efficiency was assessed through microscopic observation and imaging, combined with protoplast concentration counting using a hemocytometer (Figures 3A–D). All three enzyme systems effectively generated protoplasts; however, formulation 1 (20 mg/mL driselase, 15 mg/mL lyticase, 15 mg/mL snailase) demonstrated the highest extraction efficiency, as evidenced by morphological integrity and density of released protoplasts (Figures 3A, D).

Based on this optimal enzyme formulation, further optimization of hydrolysis time was performed to maximize both yield and quality. Samples were collected at intervals of 0.5, 1, 1.5, 2, 2.5, and 3 h for microscopic evaluation (Figures 3E–J). Protoplast counts were determined using a hemocytometer, with results presented in Figure 3K. Protoplast yield reached 1.59 × 10^7^ mL after 0.5 h of hydrolysis and increased to 5.26 × 10^7^ mL after 3 h, representing a statistically significant improvement over shorter durations (*p* < 0.05). Moreover, as illustrated in Figures 3E–J, protoplast release efficiency and morphological characteristics varied markedly with hydrolysis time. At 0.5 h, protoplasts were small and incompletely released. With progressive extension of hydrolysis time from 0.5 to 3 h, both the extent of protoplast release and their morphological quality improved substantially, with protoplasts exhibiting increasingly uniform spherical shapes. Although minor cellular debris could be observed at 3 h, the majority of protoplasts exhibited a plump morphology with smooth edges, uniform size distribution, and preserved structural integrity and suitable for transformation. Considering both protoplast yield and overall morphological quality, 3 h was selected as the optimal hydrolysis time for protoplast preparation.

### 3.3 Construction of fluorescent strains via PEG-mediated protoplast transformation

**Figure 4:**
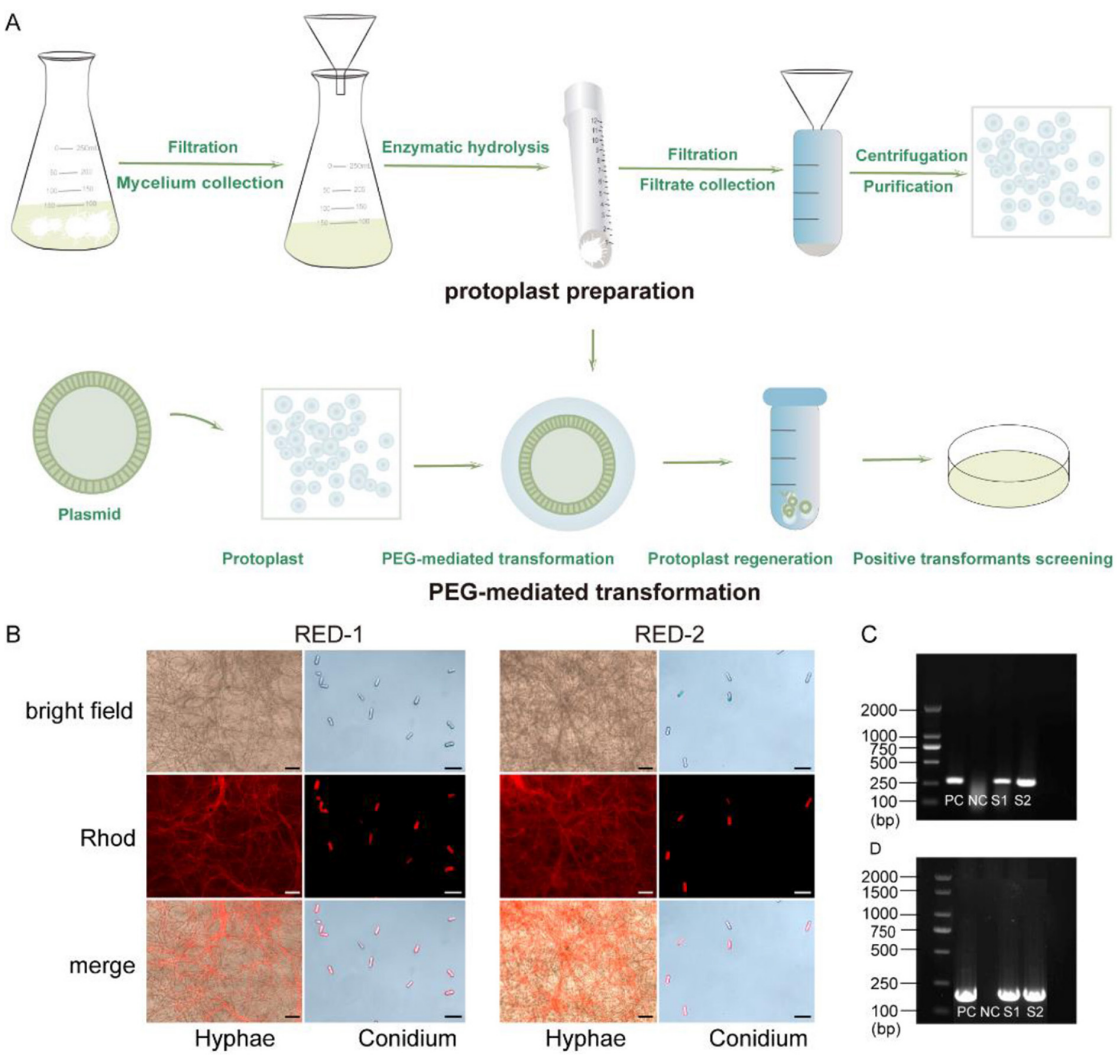
Construction of fluorescent-tagged strains. **(A)** Flow of obtaining fluorescent strains; **(B)** Fluorescence observation of conidia and mycelia from fluorescent-tagged strains; **(C, D)** PCR verification of **(C)** *neo* and *DsRED2* **(D)** in positive fluorescent-tagged strains); (PC: positive control - pKD7-RED, NC: negative control - WT). Scale bars: B = 50 μm.

*C. camelliae* F5 was used as the recipient strain, and the plasmid pKD7-RED-carrying both *neo* gene and the red fluorescent protein (RFP) coding sequence—was introduced into mycelial protoplasts via PEG-mediated transformation to generate strains expressing red fluorescence. The detailed workflow for constructing the fluorescent strain is illustrated in Figure 4A. Protoplasts were isolated through 3 h of enzymatic digestion as described in Section 2.3, purified, and resuspended in STC buffer. The protoplast concentration was adjusted to 1 × 10^8^/mL. Following transformation with pKD7-RED, transformants were selected on medium supplemented with G418. Red fluorescence was confirmed using a Zeiss fluorescence microscope, and strong fluorescent signals were detected in both conidia and hyphae (Figure 4B). Additionally, genomic DNA was extracted from putative transformants for PCR analysis to verify integration of the *neo* and *RFP* gene. As shown in Figures 4C, D, distinct amplification products of the expected sizes (*neo*: 282 bp, *DsRED2:* 219bp) were obtained from transformants RED-1 and RED-2. Corresponding bands were also observed in the positive control (plasmid pKD7-RED), whereas no amplification was detected in the wild-type (WT) negative control.

Positive fluorescent-tagged transformants were further cultured till to the fifth generation, and fluorescence was monitored in each generation. The red fluorescence was consistently observed for five consecutive generations in the fluorescent strains RED-1 and RED-2 (Supplementary Figure S1). These results demonstrated that the *neo* gene and RFP coding gene have been successfully integrated into the transformant genome, and the RFP coding gene exhibits stable expression.

### 3.4 Verification of pathogenicity and phenotypic characterization of fluorescent strains

**Figure 5:**
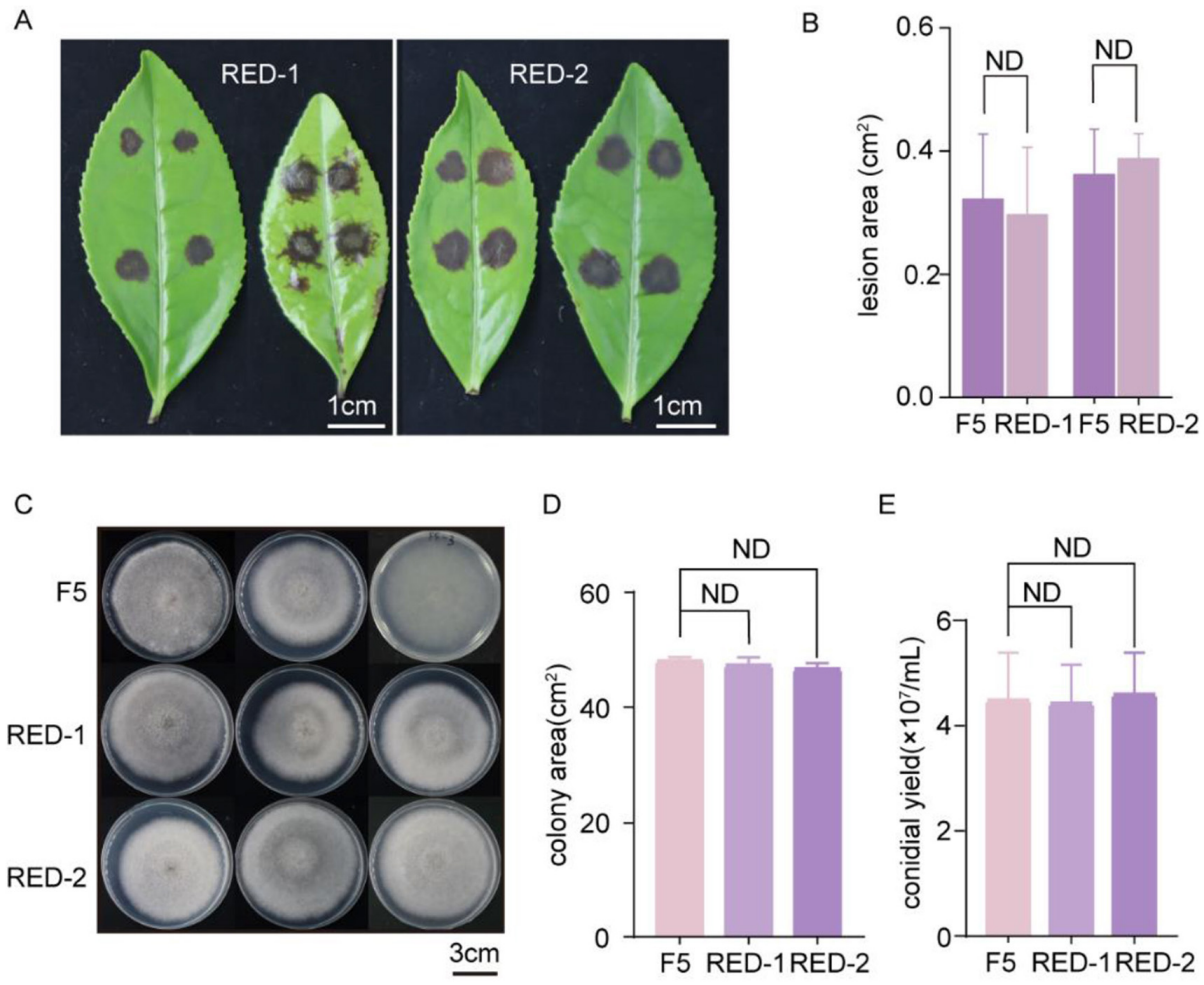
Pathogenicity assay and phenotypic characterization of fluorescent strains. **(A)** Pathogenicity assay (WT inoculated on the left side of leaf veins, and fluorescent strains on the right side); **(B)** Measurement of lesion area; **(C)** Colony morphology following 7 days of incubation on PDA medium; **(D)** Colony area quantification after 7 days of incubation on PDA medium; **(E)** Conidial yield determination using a hemocytometer (ND: no difference; two-tailed *t-*test).

To assess whether RFP expression affects pathogenicity, fluorescent transformants and WT strains were symmetrically inoculated on the abaxial surface of tea leaves and incubated under moist conditions at 28 °C. Pathogenicity was evaluated by comparing lesion development. After 2 days of incubation, visible lesions were observed in both WT and transformed strains (Figure 5A; WT inoculated on the left side of leaf veins, fluorescent strains on the right). Lesion areas were quantified using ImageJ (Figure 5B) and statistically analyzed using a two-tailed *t-*test. No significant difference in lesion size was observed between the fluorescent transformants (RED-1, RED-2) and WT (*p* > 0.05), indicating that RFP expression does not compromise the pathogenicity of *C. camelliae* F5.

Both WT and fluorescent strains were cultured on PDA medium under identical conditions to compare mycelial growth rates (Figure 5C). Colony area after 7 days of growth were measured using ImageJ (Figure 5D). Statistical analysis revealed no significant difference in growth rate between transformants and WT (*p* > 0.05). Conidiation levels are presented in Figure 5E. Collectively, these results indicate that insertion of the RFP expression cassette does not adversely affect conidial production in *C. camelliae* F5.

### 3.5 Observation of conidial germination and infection process in fluorescent strains

**Figure 6:**
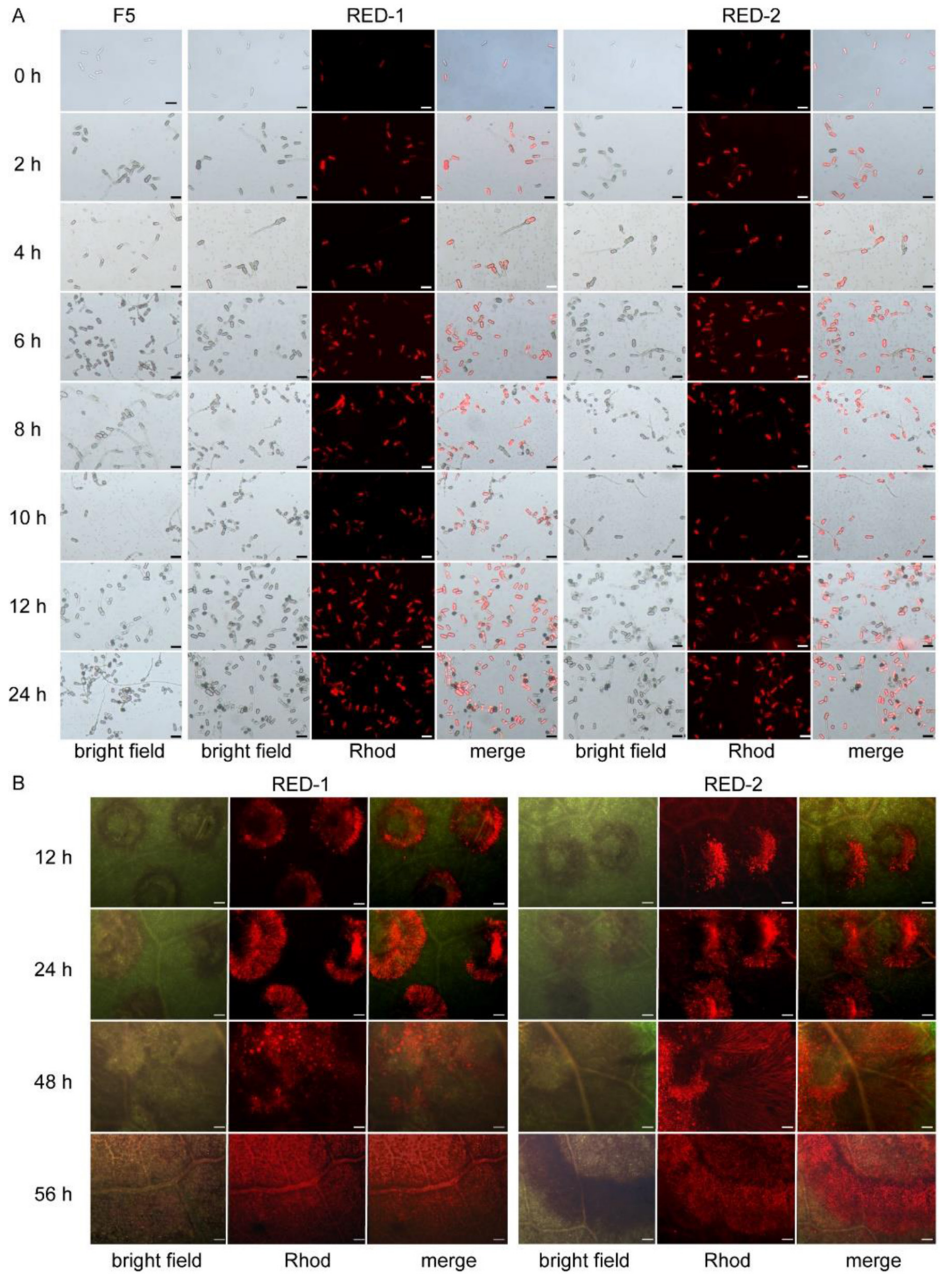
Observation of conidial germination and infection process. **(A)** Conidial germination; **(B)** Infection process. Scales bars: **(A)** = 50 μm, **(B)** = 200 μm.

As shown in Figure 6, conidial germination and appressorium induction in fluorescent strains RED-1 and RED-2 were consistent with those of the wild type. By 6 h, continued germination and swelling at germ tube tips indicated early appressorium formation. At 12 h, appressoria began to melanize, and melanization intensified by 24 h. Throughout all developmental stages, robust RFP signals were consistently detected, confirming successful transformation, transcriptional activity, and stable expression of the RFP reporter (Figure 6A).

Following inoculation of RED-1 and RED-2 onto tea leaves, the infection process was monitored. At 12 h post-inoculation, conidia and hyphae were observed adhering to the leaf surface. As infection progressed, lesion expansion was evident between 24 and 48 h. By 48 h, fungal penetration into plant tissues occurred primarily through wound sites, and clear intracellular red fluorescence was detected within host cells at 56 h post-inoculation (Figure 6B), confirming successful host invasion and colonization.

### 3.6 Application of PEG-mediated genetic transformation in targeted gene knockout

The successful generation of stably fluorescent strains confirms the establishment of an efficient PEG-mediated protoplast transformation system in *C. camelliae*. To further validate the robustness and applicability of this system, it was employed in a targeted gene knockout experiment. Gene knockout was achieved via homologous recombination, whereby the target gene was replaced with a resistance gene cassette flanked by left and right homologous arms; the detailed procedure and underlying principle are illustrated in Figure 1. In this study, the cutinase gene (designated *CU7*) was selected as the target for disruption, and the pathogenicity of the resulting transformants was subsequently evaluated. This work not only established a functional gene knockout system but also provided a foundation for investigating the role of cutinase in the pathogenesis of *C. camelliae*.

#### 3.6.1 Construction of cutinase gene (designated *CU7*) knockout fragments

**Figure 7:**
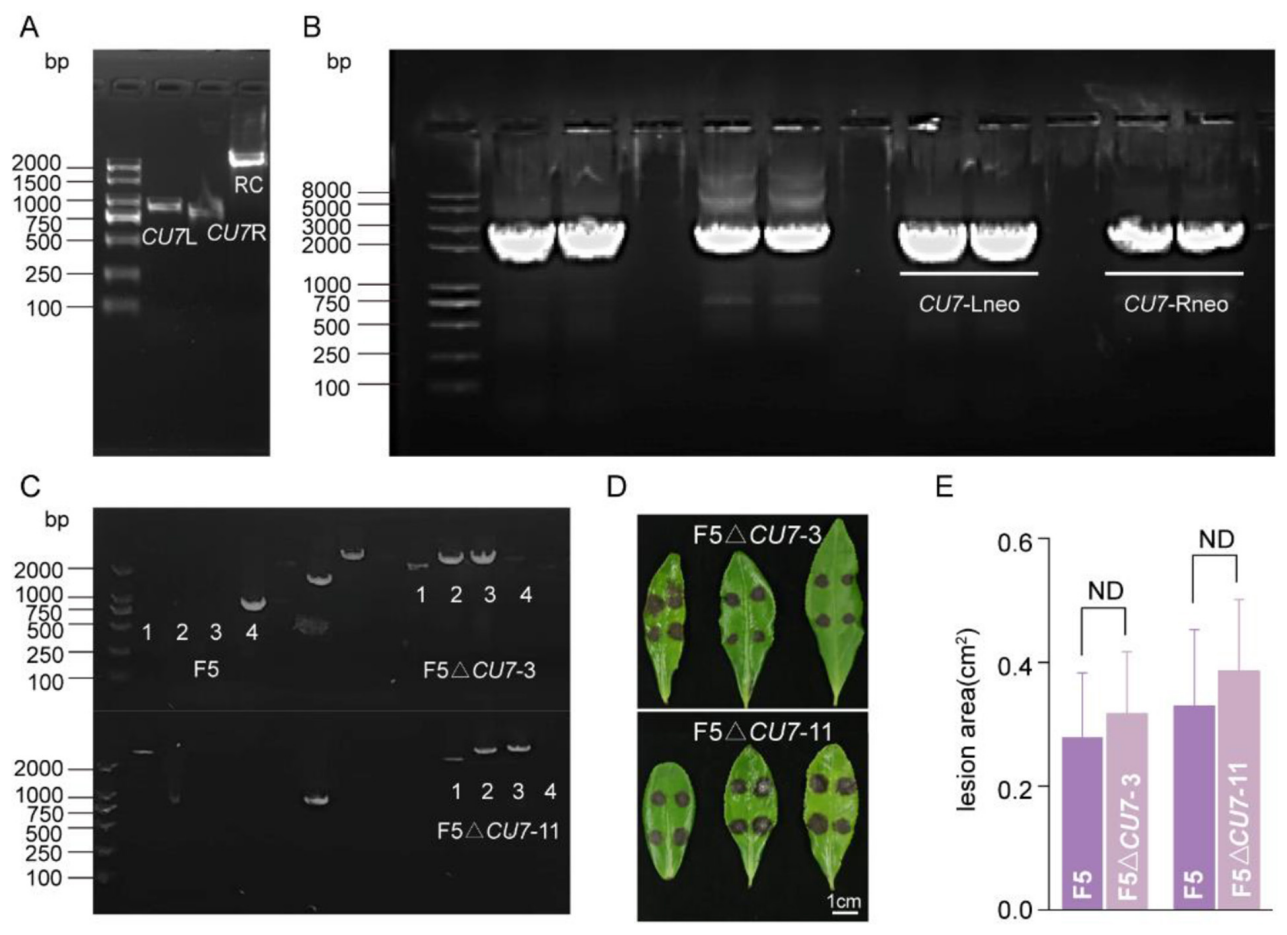
Construction and virulence validation of *CU7* gene-deleted mutants. **(A)** Amplification of *CU7* gene homologous arms and resistance gene cassette (*CU7*L: left homologous arm, *CU7*R: right homologous arm, RC: resistance gene cassette); **(B)** Knockout fragments of *CU7*; **(C)** PCR identification of transformants (1: resistance gene cassette; 2: *CU7*Lup; 3: *CU7*Rdown; 4: *CU7*); **(D)** Pathogenicity assay of *CU7* knockout mutants (wounded Inoculation: WT **(left)** / knockout mutants **(right)** of leaf veins); **(E)** Lesion area quantification in pathogenicity assays via Image J software, with statistical analysis performed using two-tailed *t-*test (ND means no difference, *p* > 0.05).

Using genomic DNA of *C. camelliae* F5 as a template, the left homologous arm (882 bp) was amplified via PCR with the primer pair sp*CU7*LF and sp*CU7*LR, while the right homologous arm (811 bp) was amplified using the primer pair sp*CU7*RF and sp*CU7*RR (Figure 7A). The *gpda-neo-trpC* resistance gene cassette (2234 bp) was amplified from the pUC_57 plasmid containing the resistance cassette sequence using primers G418F and G418R (Figure 7A). Subsequently, fusion PCR was employed to link the resistance gene cassette with the respective homologous arms. Specifically, the chimeric fragment *CU7*-Lneo (2371 bp), comprising the left homologous arm and a partial resistance gene cassette (1489 bp), was amplified using primers sp*CU7*LF and 2/3S_G418. Similarly, the *CU7*-Rneo fragment (2312 bp), consisting of the right homologous arm and a partial resistance gene cassette (1501 bp), was generated using primers 2/3X_G418 and sp*CU7*RR. The resulting knockout fragment was confirmed by agarose gel electrophoresis (Figure 7B), purified through gel extraction, and further validated by sequencing. The sequence accuracy was verified, and the fragment was subsequently prepared for protoplast-mediated transformation. Primer sequences are listed in Table 1.

**Table 1:** Primers for gene knockout.

Primer	Primer sequence (5^′^-3^′^)
sp*CU7*LF	GCCCAACCACCCACCTCTAA
sp*CU7*LR	GAGTCACCGGTCACTGTACATCTTCCGTGACGTGCAACTAG
sp*CU7*RF	GACSATGGAGCTATTAAATCATCCTTCTTCCCTTTCAACCTTT
sp*CU7*RR	GATGCCGATTTCAACTACTACTCA
G418F	TGTACAGTGACCGGTGACTC
G418R	TGATTTAATAGCTCCATGTC
2/3SG418	GAAACGACCGTTCTCCACCA
2/3XG418	CCAAGAACCTTTATTTCCCC
*CU7*Lup-F	TACAAACCTGCGAAGTCAT
*CU7*Lup-R	TGCTCCGTAACACCCAAT
*CU7*Rdown-F	GGCAGTAAGCGAAGGAGAATGTG
*CU7*Rdown-R	GGCTATGGAGATGGGAGATGTTTT
*CU7*-F	ATGAAGGCCGGTCTCCTC
*CU7*-R	TTACTGGACGAGTCCAGCAACG

#### 3.6.2 Generation of cutinase gene *CU7* knockout mutants

The purified knockout fragments (2 μg per fragment) were introduced into protoplasts via PEG-mediated transformation, following the protocol outlined in Section 2.4. After protoplast regeneration, the colonies were overlaid on PDA medium containing 80 μg/mL G418 for preliminary screening of positive transformants. Putative positive transformants were further analyzed by PCR using mycelial DNA as template. Amplification was performed for the following targets: (i) the full-length resistance gene cassette (2234 bp) using primers G418F and G418R; (ii) the junction fragment spanning the left homologous arm and the resistance cassette (*CU7L*up, 3001 bp) using primers *CU7*Lup-F and *CU7*Lup-R; (iii) the right flanking region including the resistance cassette and right homologous arm (*CU7*Rdown, 2940 bp) using primers *CU7*Rdown-F and *CU7*Rdown-R; and (iv) the target *CU7* gene (856 bp) using primers *CU7*-F and *CU7*-R. Primer details are provided in Table 1. PCR results are presented in Figure 7C. While the *CU7* gene was successfully amplified in the WT strain, no amplification was observed in the transformants, indicating successful gene replacement. Transformants yielding both *CU7*Lup and *CU7*Rdown junction fragments (F5Δ*CU7*-3 and F5Δ*CU7*-11) were confirmed as correct gene replacement events.

#### 3.6.3 Pathogenicity assessment of *CU7* knockout mutants

To evaluate the functional role of *CU7* in the pathogenicity of *C. camelliae* F5, knockout strains F5Δ*CU7*-3 and F5ΔCU7-11 were inoculated onto the abaxial surface of tea leaves using a wounding method, with the WT strain serving as control. Inoculations were conducted symmetrically to allow direct comparison. Lesion development was monitored and documented (Figure 7D). Lesion areas were quantified using ImageJ software (Figure 7E), and statistical analysis was performed using an independent samples *t-*test in SPSS. No significant difference in lesion size was observed between the mutant strains and the WT (*p* > 0.05). These findings indicate that deletion of the cutinase gene *CU7* does not impair the pathogenicity of *C. camelliae* F5 under wounded inoculation conditions.

## 4 Discussion

*C. camelliae*, the primary species responsible for anthracnose in tea plants, has garnered significant attention due to its strong pathogenicity and high isolation rate (Wang et al., 2016; Peng et al., 2023). In recent years, there has been an increasing focus on the functional genomics of phytopathogenic fungi. The sequencing and availability of the *Colletotrichum* genome have considerably enhanced our understanding of the fundamental mechanisms underlying its pathogenicity and infection processes, thereby establishing a foundation for the development of effective control strategies against tea plant anthracnose (Chen et al., 2022; Wang et al., 2023; Kong et al., 2023). Given that functional characterization of genes and validation of their roles often necessitate genetic manipulation, we report an efficient PEG-mediated transformation approach for genetic modification—including insertional mutagenesis and targeted gene disruption—in *C. camelliae*.

The efficiency of protoplast preparation from filamentous fungi is a critical determinant of successful genetic transformation. The cell walls of filamentous fungi are typically thick and structurally complex, primarily composed of chitin, glycoproteins, lipids, and glucans (Yang, 2011). Due to variations in cell wall composition across species, tailored enzyme mixtures are required to effectively degrade the cell wall and generate viable protoplasts. Thus, the selection of appropriate lytic enzymes and optimization of hydrolysis conditions represent pivotal steps in protoplast isolation. Previous studies have demonstrated that mixed enzyme systems achieve superior cell wall lysis compared to single-enzyme treatments (Hu et al., 2015; Chen et al., 2011). In this study, a combination of driselase, lyticase, and snailase was employed for enzymatic digestion, resulting in significantly enhanced protoplast efficiency relative to previously reported protocols (Lv et al., 2023).

Following cell wall removal, exogenous DNA can be introduced into fungal protoplasts via PEG-mediated transformation. Once internalized, the foreign DNA rapidly associates with host cellular proteins to form a stable complex, which protects it from nuclease degradation. This protein-DNA complex is subsequently transported into the nucleus through the nuclear pore complex, where it may integrate into the genomic DNA (Lichius et al., 2020). Integration of exogenous DNA in filamentous fungi can occur either randomly throughout the genome or at specific loci via homologous recombination (Lichius et al., 2020). In this work, fluorescently labeled strains were generated through random integration of exogenous DNA into the *C. camelliae* F5 genome, marking the successful establishment of a robust genetic transformation system for this organism.

The development of such a transformation platform provides essential technical support for reverse genetics approaches, including gene knockout and functional gene analysis. In our genetic manipulation of *C. camelliae*, we implemented the split-marker deletion strategy—originally developed in *Saccharomyces cerevisiae*—which separates the homology arms from the selectable marker cassette. This design minimizes self-ligation of linear DNA fragments during transformation and substantially enhances the frequency of targeted integration. By integrating the principles of homologous recombination, utilizing geneticin as a selection agent, and leveraging the PEG-mediated protoplast transformation system, we achieved precise replacement of the target gene with a resistance gene cassette, thereby accomplishing targeted gene knockout (Fairhead et al., 1996, 1998). Notably, this study demonstrates strategic differentiation in gene integration methods: while gene knockout relies on homologous recombination, the construction of fluorescent reporter strains exploits the non-homologous end joining (NHEJ) pathway, leading to random genomic integration of the fluorescent protein gene in *C. camelliae* F5 (Lichius et al., 2020). The successful implementation of both integration mechanisms not only highlights the versatility of the established genetic system but also confirms the efficacy of using protoplasts as recipients for achieving either site-specific or random integration of exogenous DNA in *Colletotrichum* species.

Moreover, the PEG-mediated transformation system enables high-throughput functional analysis of multiple genes, facilitating investigations into the molecular mechanisms of pathogenesis. The plant cuticle constitutes the primary physical barrier against microbial invasion. Pathogenic fungi may breach this barrier by entering through natural openings such as stomata, directly penetrating the cuticle layer, or secreting cutin-degrading enzymes to facilitate host entry (Kolattukudy, 1985; Kolattukudy et al., 1995). Considering the pivotal role of cutinase in fungal infectivity, we selected a member of the cutinase gene family for functional disruption. Through the combined application of PEG-mediated transformation and the split-marker technique, we successfully knocked out the target gene, providing a foundation for further elucidation of its role in the infection process.

When the cutinase gene knockout mutants were inoculated onto tea leaves using wounded inoculation, their pathogenicity was found to be comparable to that of the WT. (Auyong et al. 2015) demonstrated through RNA interference (RNAi) technology that the functional deficiency of the cutinase gene *CtCut1* in *C. truncatum* leads to a significant reduction in fungal pathogenicity. However, following deliberate injury to the host cuticle, the defective strain successfully infected and colonized the host, indicating that *CtCut1* is essential for *C. truncatum* to penetrate the cuticle of pepper fruits and achieve successful infection (Auyong et al., 2015). In our study, the unaltered pathogenicity of the cutinase gene knockout mutants compared to the WT under wounded inoculation conditions may be attributed to the compromised tea leaf cuticle, which facilitates the strains' invasion through mechanical wounds. Notably, a wound-free inoculation system for tea plants has not yet been established, underscoring the necessity of future investigations into the potential role of the cutinase gene in mediating cuticle penetration by *C. camelliae*. The potential objective of future studies is to establish a non-wounding infection model for *C. camelliae* on tea plants, which would facilitate a comprehensive understanding of the cutinase gene's role during host invasion.

In summary, we successfully established a PEG-mediated genetic transformation system and applied it to generate fluorescent strains and cutinase gene deletion mutants, thereby facilitating investigations into the fungal invasion process and providing insights for future functional studies of the cutinase gene. Furthermore, this transformation system offers a stable technical platform for comprehensive functional analyses of key genes in *C. camelliae*, including those involved in pathogenicity and metabolic regulation. It may also serve as a methodological reference for molecular genetic manipulation of related filamentous fungi.

## Data Availability

The original contributions presented in the study are included in the article/Supplementary material, further inquiries can be directed to the corresponding author/s.
