# Supplementary material for: PEG-mediated transformation: a tool for random integration or targeted gene replacement in Colletotrichum camelliae
Source: Front Microbiol. 2026 Apr 24;17:1814272. doi: 10.3389/fmicb.2026.1814272 (PMC13154153; doi:10.3389/fmicb.2026.1814272)
Supplement: Supplementary file 1 [file Supplementary_File_1.docx]

Supplementary materials

Figure S1 Microscopy observation of florescent transformants. A: RED-1; B: RED-2. Scale bars: A-B=20 µm.

| 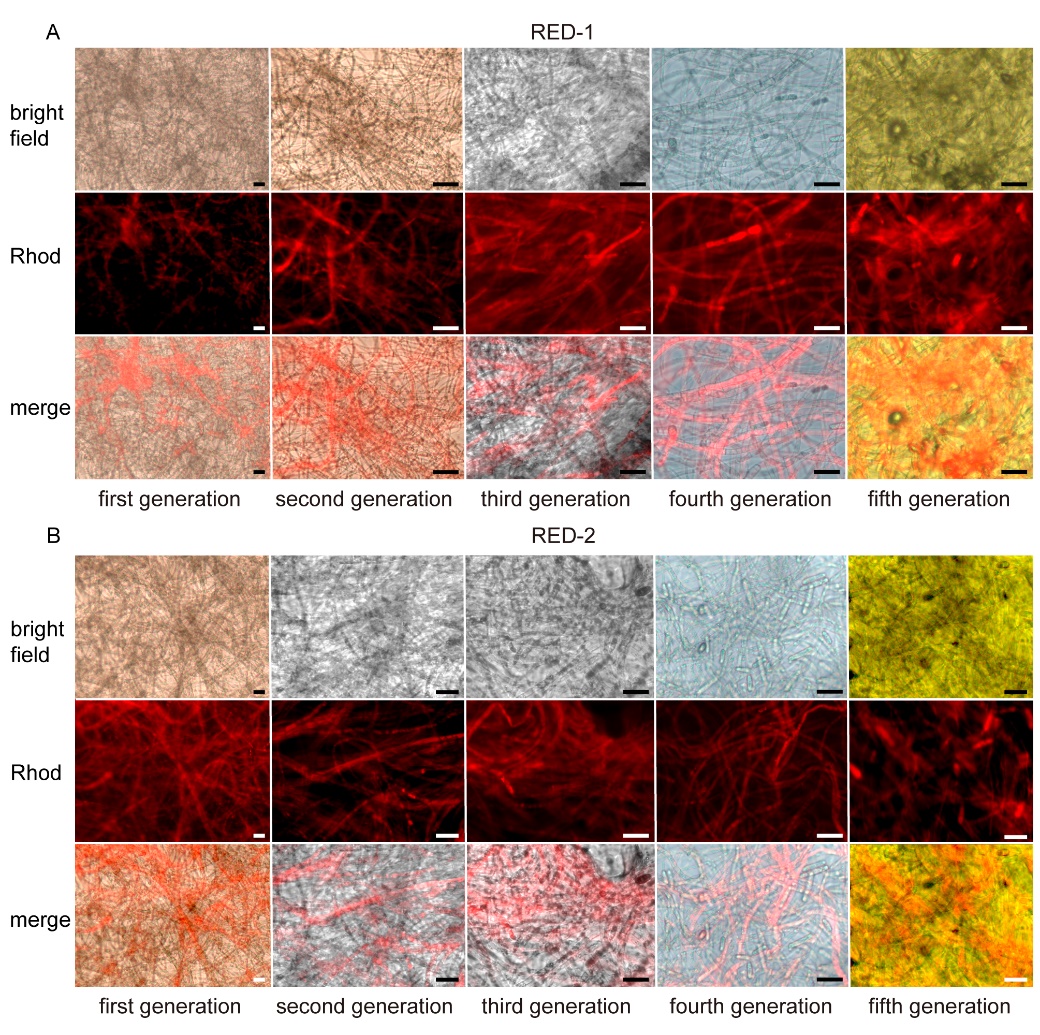 |
| --- |
| Figure S1 |
